# Recovery of fitness of a live attenuated simian immunodeficiency virus through compensation in both the coding and non-coding regions of the viral genome

**DOI:** 10.1186/1742-4690-4-44

**Published:** 2007-07-03

**Authors:** James B Whitney, Mark A Wainberg

**Affiliations:** 1 McGill University AIDS Centre, Lady Davis Institute-Jewish General Hospital, Montreal, Quebec, H3T 1E2, Canada; 2 Department of Microbiology and Immunology, McGill University, Montreal, Quebec, H3A 2B4, Canada; 3 Division of Viral Pathogenesis, Beth Israel Deaconess Medical Center, Harvard Medical School, Boston, MA 02115, USA

## Abstract

We have analyzed a SIV deletion mutant that was compromised both in viral replication and RNA packaging. Serial passage of this variant in two different T-cell lines resulted in compensatory reversion and the generation of independent groups of point mutations within each cell line. Within each group, single point mutations were shown to contribute to increased viral infectivity and the rescue of wild-type replication kinetics. The complete recovery of viral fitness ultimately correlated with the restoration of viral RNA packaging. Consistent with the latter finding was the rescue of Pr^55 ^Gag processing, also restoring proper virus core morphology in mature virions. These seemingly independently arising groups of compensatory mutations were functionally interchangeable in regard to the recovery of wild type replication in rhesus PBMCs. These findings indicate that viral reversion that overcomes a genetic bottleneck is not limited to a single pathway, and illustrates the remarkable adaptability of lentiviruses.

## Background

The packaging of full-length viral genomic RNA (vRNA) into primate lentiviruses is regulated by a multipartite *cis*-acting signal located within the 5' untranslated region (UTR) or RNA-leader. In the leader of human immunodeficiency virus type-1 (HIV-1), the packaging signal or Psi (Ψ) is distributed across multiple RNA domains that include stem loop-1 (SL1), SL3 and SL4 [1-3]. There is also evidence of vRNA packaging elements in other regions, including those upstream of the primer-binding site (PBS), as well as within downstream *gag*-coding regions [4,5].

Comparative packaging studies of simian immunodeficiency virus (SIV) by our group and of human immunodeficiency virus type-2 (HIV-2) by others, have assigned a primary role in packaging to SL1, as compared to all other regions within the SIV and HIV-2 genomes [6-10]. Moreover, SL1 sequences are also important in the formation of 5' linked vRNA duplexes or vRNA dimers [8,11-13]. RNA-RNA interactions ultimately determine RNA tertiary conformation and have been shown to impact on both the regulation and efficiency of vRNA packaging [14,15]. The relationships among the packaging events of different lentiviruses have been extensively studied [16].

The foregoing implies the presence of multiple RNA-binding domains within Pr^55 ^Gag. In the context of Pr^55 ^Gag an important trans-role has been ascribed to the viral nucleocapsid (NC) protein [17,18], although several studies have indicated that a functional separation of domains within NC is present [17,19]. Other protein domains within Gag have also been shown to be necessary for vRNA packaging and dimerization, whereas the p2 region has been shown to contribute to vRNA packaging specificity [20,21].

Studies on the reversion of SL1 deleted virus in HIV-1 showed that compensatory point mutations in four distant Gag proteins, i.e. nucleocapsid (NC-T24I), matrix (MA-V35I), capsid (CA-T24I) and the p2-spacer (p2-T21I) were all involved in restoration of viral growth [22]. Griffin et al have shown that there is a preferential use of co-translation to impart packaging specificity for vRNA in HIV-2 [23]. A similar process is thought to occur in SIV, particularly in light of evidence that the 3' regions of the leader possess an internal ribosome entry site (IRES) function [24].

Although Pr^55^Gag alone has been shown to be sufficient for particle production, numerous host and viral proteins are required for optimal viral assembly and budding [25]. Indeed, an appropriate conformation of packaged RNA is critical, since mutations in viral RNA can severely impact virus production and viability [26]. The late phase of lentiviral replication requires the assembly of virion components at the cellular periphery, at which a series of interrelated vRNA-protein interactions are required to occur in a coordinated fashion; this positions vRNA in precise relation to Pr^55^Gag during protease-mediated cleavages that take place during assembly and at post-budding stages [27].

Previous work from our group described a mutant deleted of 21 nucleotides within the 5' proximal stem of SL1 of the infectious molecular clone of SIV_mac239 _(Δnt +398 to +418, termed-SD2) that resulted in a significant delay in viral replication and reduced vRNA packaging. The serial passage of this mutant virus in the CEMx174-T/B-hybrid cell line or in C8166-T cells over protracted periods resulted in the recovery of virus replication [7]. Our previous report showed that the original SD2 deletion had been retained, but that each cell line specific isolate harboured three additional compensatory point mutations.

**Figure 1:**
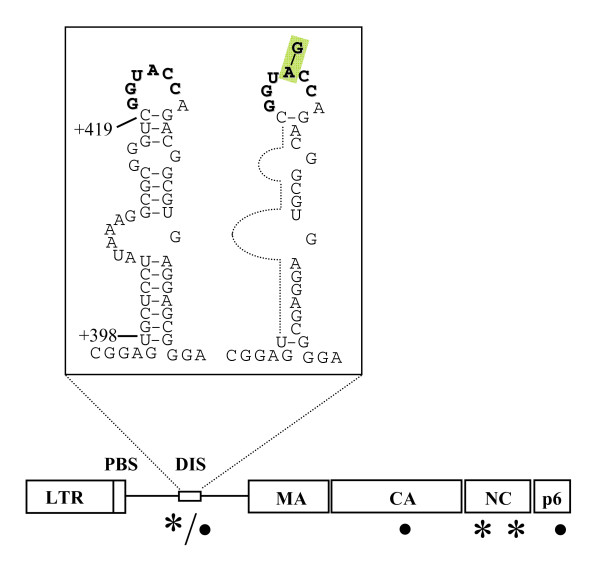
**RNA secondary structure of SL1 and position of the SD2-nucleotide deletion in the SIV leader**. Secondary structure of the SIV_mac239 _SL1 RNA element was predicted by free energy minimization and adapted from published information [6, 28, 48]. All nucleotide deletions are relative to the transcriptional initiation site (1+) based on the sequence of the wild type clone of SIV_mac239_. The DIS palindrome is shown in bold, the A423G compensatory mutation is highlighted. Below is a diagram of the location of the various compensatory mutations generated in different cell lines. **Asterisks **denote substitutions selected in CEMx174 cells, **Bullets **denote substitutions selected in C8166 cells.

Briefly, virus passaged in C8166 cells, a single A-G compensatory point mutation was identified within the viral dimerization initiation site (DIS) at nucleotide position +423 (A423G), while two other compensatory mutations were found in the CA and p6 regions of gag, (i.e. K197R and G49K, respectively) [22]. The forced evolution of the SD2 variant in CEMx174 cells also selected the A423G substitution. However, two distinct mutations were also identified in NC, i.e. E18G and G31K (Fig. 1).

The present study was designed to elucidate mechanisms whereby various compensatory mutations can restore viral replicative fitness, and the role of different cellular environments on the molecular evolution of SIV genomes harboring deletions in leader sequences. We now show that the recovery of Pr^55^Gag protein processing is commensurate with the return of wild-type levels of packaged vRNA. We also show that some mutations can facilitate partial recovery of RNA dimerization, leading to restored viral core morphology and placement. Thus, compensation may involve different viral gene products, leading to restored infectivity and replicative fitness in primary PBMCs.

## Results

### The A423G point mutation plays an important role in the restoration of viral RNA packaging

The SD2 variant (Δnt +398 to +418, Fig. 1) has been shown to package diminished levels of viral RNA in comparison with wild type SIV_mac239_[6,7]. Forced evolution of the SD2 variant through serial passage resulted in the restoration of wild-type replication kinetics. To further investigate the mechanism(s) involved, seven different SD2 derivates were analyzed that contained all possible combinations of the three point mutations that had been identified in cell lines [7]. Viruses that reverted in C8166 cells contained either one, two or all three of the above mentioned mutations, as follows: SD2-A423G, SD2-K197R, SD2-G49L, SD2-A423G, K197R, SD2-A423G, G49K, SD2-K197R, G49L, and SD2-A423G, K197R, G49K. Similarly, viruses derived from reversions in CEMx174 cells were termed SD2-A423G, SD2-E18G, SD2-G31K, SD2-E18G, E31K, SD2-A423G, E18G, SD2-A423G, E31K, and SD2-A423G, E18G, G31K (Fig. 1 and Table 1).

**Table 1:** Impact of various coding and non-coding compensatory mutations on SD2 fitness.

	**Mutation**	**Location**	**Charge**	**Processing**	**Infectivity**	**RNA incorporation**
CEMx174	A420G	DIS-SL	n/a	n/c	+++	+++
	E18G	NC	+	+++	+	+
	G31K	NC	+	+++	++	++
C8166	A420G	DIS-SL	n/a	n/c	+++	+++
	K197R	CA	n/c	n/c	++	+
	G49K	P6	+	+++	++	+

Legend: + = moderate recovery, +++ = near complete recovery, n/a = not applicable, n/c = no change

Viral DNA of each of the two mutant groups (i.e. generated in either C8166 or CEMx174 cells) were transfected into 293T cells. Mutant viral RNA was extracted from aliquots of the supernatants of these transfections and normalized on the basis of p27-CA. To assess relative packaging efficiency, mutant viral RNAs were used as template in an 18-cycle multiplex RT-PCR reaction run in parallel with multiple dilutions of wild type vRNA as a linear range control, as described previously [6].

**Figure 2:**
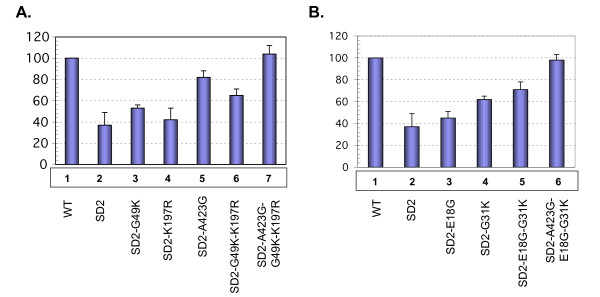
**Effects of untranslated-leader and gag-coding region mutations on viral RNA encapsidation**. Equivalent amounts of virus derived from transfected 293T cells, based on levels of p27-CAantigen, were used to prepare viral RNA that was then used as template for quantitative RT-PCR to detect full-length viral RNA genome in an 18-cycle PCR reaction [6]. Relative amounts of a 114-bp DNA product were quantified by molecular imaging, with wild-type values arbitrarily set at 1.0. Reactions run with RNA template, digested by DNase-free RNase, served as a negative control for each sample to exclude any potential DNA contamination. Relative amounts of viral RNA that were packaged were determined on the basis of four different experiments. **A**. RT-PCR vRNA packaging results of SD2 variants harboring compensatory mutations in the DIS (A423G), CA (K197R) and p6 (G49K) regions. **B**. RT-PCR vRNA packaging results of SD2 variants harboring mutations in the DIS (A423G), and NC (E18G and G31K) regions.

The results of RT-PCR (Fig. 2), were subjected to DNA imaging analysis that showed that the SD2 deletion mutant packaged viral RNA at levels that were approximately 40% of wild type; this is in agreement with previous studies. The compensatory A423G mutation within the DIS-SL yielded the single largest increase in packaging efficiency to about 80% of wild-type levels. In contrast, the SD2-G49K and SD2-K197R variants packaged only very low levels of vRNA (Fig 2A). Combinations of the K197R and G49K mutations, i.e. SD2-K197R, G49K, or of all three mutations, i.e., SD2-A423G, K197R, G49K, showed increased packaging efficiency.

Next, we tested the ability of the two NC mutations to restore vRNA incorporation. Fig 2B shows that the presence in SD2 of either E18G or G31K alone only marginally affected levels of viral RNA packaging. In contrast, the presence of both NC mutations resulted in moderately increased RNA packaging. The addition of the A423G mutation to the construct that contained both NC substitutions completely compensated for the packaging deficit.

The SD2 variant (Δnt +398 to +418, Fig. 1) has also been shown to be devoid of an RNA dimer [6-8]. To determine the impact of multiple compensatory mutations on vRNA dimerization, we analysed purifed vRNA on non-denaturing Northern gels.

**Figure 3:**
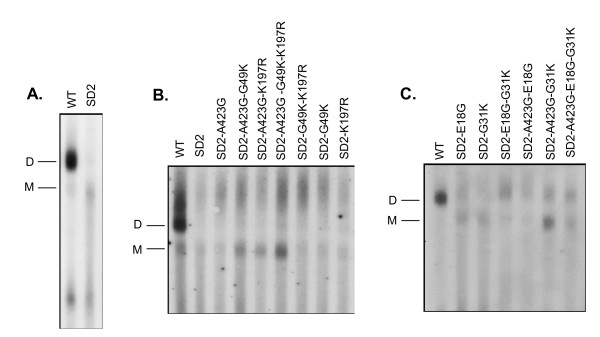
**Native analysis of virion-associated RNA**. Mutant or wild-type virus was purified by sucrose gradient ultracentrifugation. Virion RNA was then extracted from lysed particles by protease K digestion followed by phenol chloroform extraction. RNA was run under non-denaturing conditions at room temperature. Membranes were analyzed with an SIV specific probe as described in Materials and Methods. A. Non-denaturing Northern analysis of the SD2 variant in conjunction with compensatory mutants in the DIS, CA, and p6. B. Non-denaturingNorthern analysis of the SD2 variant in conjunction with compensatory mutants in the DIS and in the NC protein.

The results (Fig. 3A) show that RNA preparations recovered from the SD2-mutants are compromised in regard to vRNA dimerization compared to native wild-type RNA. Figure 3B shows that the addition of the A423G mutation to the SD2 backbone increased vRNA encapsidation levels but appeared to have little impact on the amount of packaged, mature vRNA dimer. Similarly, the amount of mature dimer was not influenced by the addition of the G49K or K197R mutations, i.e. SD2-G49K or SD2-K197R (Fig. 3B). However, each of the abovementioned variants did result in increased levels of high mobility RNA, interpreted to be dimeric RNA in an immature state, on non-denaturing gels. This was also observed for combinations of the K197R and G49K mutations, i.e. SD2-K197R, G49K, or of all three compensatory mutations, i.e., SD2-A423G, K197R, G49K.

Next, we tested the ability of the two NC mutations to rescue vRNA dimerization. Figure 3C shows that the SD2 variant did have slightly increased levels of dimeric RNA in the presence of either E18G or G31K. In contrast, the presence of both NC mutations resulted in a moderate increase in levels of dimeric RNA. The addition of the A423G and E18G mutations to the SD2 parental strain also yielded an increase in RNA dimer levels. Finally, the addition of G31K or of both NC substitutions to the SD2-A423G variant increased levels of both packaged RNA monomer and dimer.

To shed further light on the mechanisms involved, we performed a thermodynamic RNA structural analysis of these mutants by using M-Fold software [28]. RNA secondary structure analysis suggests that the A423G point mutation, that is located in the DIS-SL loop, cannot restore native DIS-SL structure. However, our analysis indicated that the A423G mutation altered the size of the DIS-loop through nucleotide reorganization and loss of SL2 structure (not shown). Hence, the A423G point mutation plays an important role in the compensation of the SD2 deletion, but a full correction of packaging requires the presence of three mutations.

### The G49K point mutation within p6 or, alternatively, the E18G and G31K mutations within NC can restore Pr^55^Gag processing in viruses that harbour the SD2 deletion

**Figure 4:**
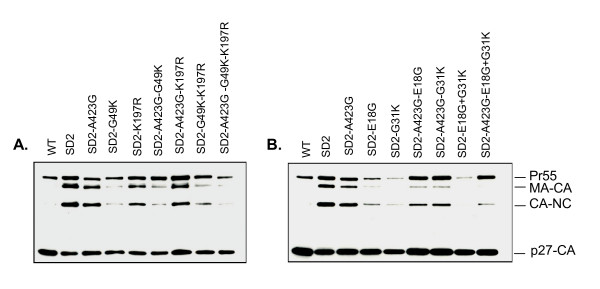
**Restoration of proteolytic Gag-processing by G49K, or by the E18G, G31K mutations**. Viruses were purified by ultracentrifugation of clarified culture supernatants over a sucrose cushion at 48h after transfection. Western analysis of viral Pr^55 ^Gag products were detected using MAb directed against p27-CA antigen.

The SD2 deletion also resulted in delayed processing and an altered processing pattern of Gag proteins. To study the role of the aforementioned compensatory mutations in this regard, Pr^55^Gag processing was evaluated by SDS-Page analysis of viral proteins and Western blotting was performed using monoclonal antibodies (MAbs) directed against p27-CA as described previously [8]. Indeed, the processing of each of three Gag proteins, i.e., the precursor protein Pr55, the intermediate proteins p41, and p39, were all impaired in the SD2 variant, but not in wild-type virus. Interestingly, we found that all viruses that contained the G49L mutation in p6, i.e SD2-G49K, SD2-K197R, G49K, SD2-A423G, G49K, and SD2-A423G, K197R, G49K, possessed similar proportions of these products as wild-type virus. In contrast, the SD2-K197R, SD2-A423G, and SD2-A423G, K197R viruses displayed an accumulation of Pr55, p41, and p39 and diminished levels of p27, similar to the parental SD2 virus (Fig. 4A).

The results of Fig. 4B show that either the E18G or G31K substitution in NC was independently able to facilitate complete Pr^55^Gag processing in the SD2 virus. In the presence of the A423G mutation, however, both NC mutations i.e., SD2-A423G, E18G, G31K, were required to restore processing of both the MA-CA (p41) and CA-NC (p39) intermediate processing products, leading to a wild type processing phenotype.

Thus, the A423G point mutation acts to rescue the deficit in viral RNA packaging of the SD2 deletion, while the G49K mutation in p6 or the E18G and G31K substitutions in NC contribute to the restoration of Gag processing.

### Both sets of compensatory mutations are functionally interchangeable in recovery of viral replication and infectivity

**Figure 5:**
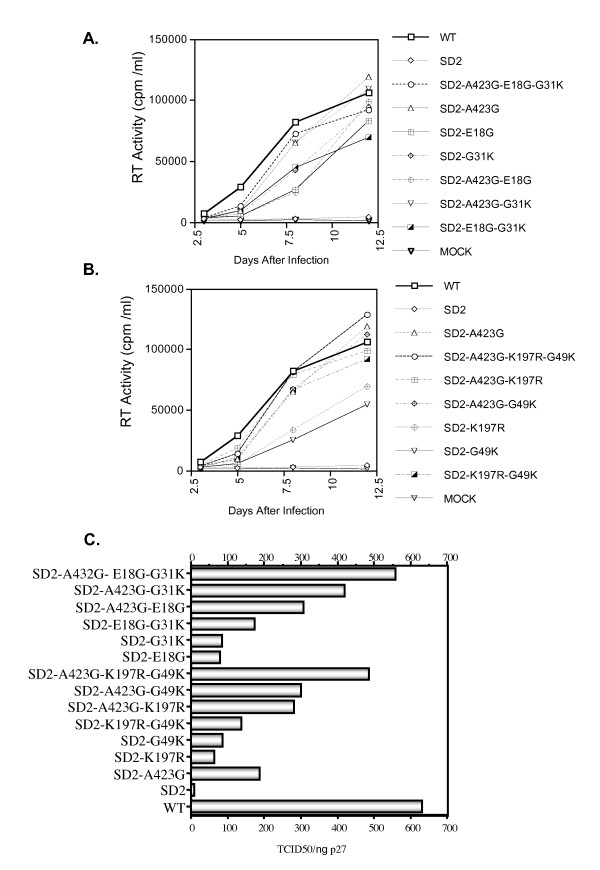
**Replicative fitness of wild-type and mutated viruses in monkey PBMCs**. Viral replication was assessed in PHA-activated rhesus PBMCs using 10ng of viral inocula normalized on the basis of p27-CA Ag. All replication experiments were conducted in triplicate. Viral replication was monitored by RT assay of culture supernatants at multiple time points. All RT activity results are the average of duplicates. Mock infection denotes exposure of cells to heat-inactivated wild-type virus as a negative control. **A**. Growth curves of SD2-variants harboring mutations in the DIS (A423G) and NC (E18G and G31K) regions. **B**. Growth curves of variants harboring compensatory mutations in the DIS (A423G), CA (K197R) and p6 (G49K) regions. **C**. Viral replication analysis of mutated viruses by TCID_50 _analysis of viral infectivity as described in Materials and Methods. Results shown are representative of three independent endpoint dilution assay experiments. The scale of the ordinate is logarithmic. Mock infection represents a negative control in which cells were exposed to heat-inactivated wild-type virus.

In order to pursue the biological relevance of these compensatory mutations, each mutant proviral construct was transfected into 293T cells and viral supernatanst harvested after 48 hours. Viral stocks were titrated by p27-CA ELISA and assayed for viral replication capacity in PHA-stimulated rhesus PBMC. As shown in Figure 5A, the mutations that had emerged in CEMx174 cells, i.e. A423G, E18G, G31K, were also able to rescue the defective replication of the SD2-deleted viruses in these primary cells. Although each single mutation could individually contribute to recovered viral growth, full restoration of replication capacity required the combination of all three mutations, i.e. SD2-A423G, E18G, and G31K. Similarly, the combination of A423G, K197R, and G49K in the same SD2-backbone fully rescued SD2 replication in rhesus PBMC (Fig. 5B).

The role of these various compensatory mutations in viral replicative fitness was next assessed on the basis of viral infectiousness in CEMx174 cells. For this purpose, relative p27-CA concentrations in viral supernatants at the peak of viral replication (as determined by RT assay and observed cytopathicity in culture) were used to calculate TCID_50 _per ng p27-CA antigen (Fig. 5C). The results show that the SD2 mutant was severely compromised, whereas each compensatory mutation was independently capable of restoring some degree of viral infectiousness, with the largest increase attributable to A423G. However, recovery to near wild-type replication levels required a full complement of either of the two groups of compensatory mutations. The mutations identified in the C8166 cell line restored infectiousness equally well when assayed in the CEMx174 line and vice versa (not shown). Thus, both sets of compensatory mutations seem to be functionally interchangeable in regard to restoration of viral replication, independent of the cell line in which they were first selected.

### Forced evolution results in restoration of proper viral core ultra-structure

**Figure 6:**
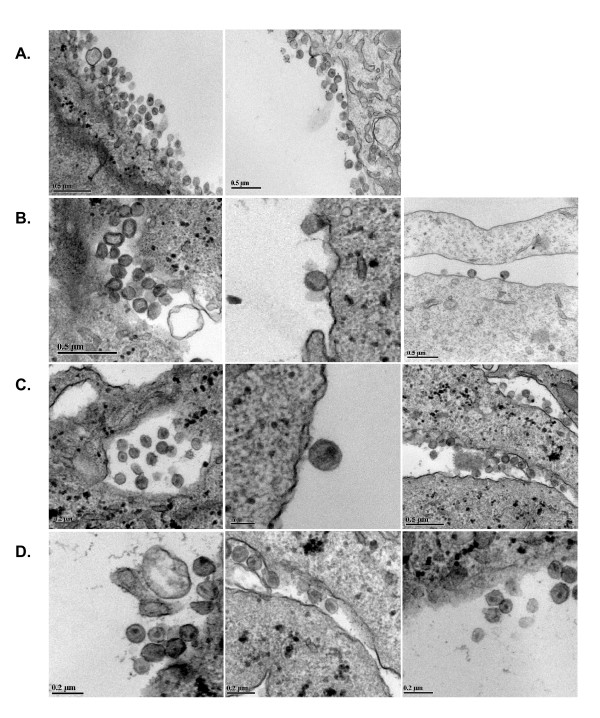
**Transmission election microscopy of wild-type and mutant viral particles**. TEM of late (fixed 48 hr post-transfection) wild-type and mutant particles were assessed and scored from multiple sections. Panel A: the wild-type virus displayed typical size and conical core morphology. Panel B: the SD2 deletion mutant showed diminished production of viral particles, with altered diameter and core morphology. Panel C: the SD2-A423G-E18G-G31K mutant showed restoration of proper core morphology. Panel D: the SD2-A423G-K197R-G49K mutant also showed restoration of core placement and morphology. Bar size is shown for each panel.

We next hypothesized that the mutations selected through serial passage might also correct morphological anomalies in the viral core. Transmission electron microscopy (TEM), of ultra-thin sections of transfected cell preparations showed that approximately 80% of wild-type virus particles contained a fully condensed core, typical of mature virus. In contrast, the SD2 mutant resulted in diminished viral production, and about 70% of the SD2 particles observed possessed displaced and/or improperly condensed cores and/or immature core structure (Fig. 6).

Both recombinant clones (i.e. SD2-A423G-E18G-G31K and SD2-A423G-K197R-G49K) were also transfected in parallel, and yielded comparable levels of particle production as wild type, as measured by p27-CA levels in culture supernatants (not shown). The results of the EM experiments showed restoration of proper core morphology, and levels of immature virus were comparable to the wild type (Fig. 6).

## Discussion

Here, we describe an SIV deletion mutant that was passaged in two different T-cell lines and that employed two different pathways to attain reversion. Retroviruses display genomic plasticity, and sequence diversification in both HIV-1 and SIV can in some cases augment viral replication and pathogenesis [29-31]. The fitness of an RNA virus population may be viewed as a continuum of genomes of varying fitness. It is not surprising that these viruses may be able to employ diverse routes to reach higher fitness levels. However, such transitions may be delimited by the tolerance of a particular gene for non-synonomous mutation versus the maintenance of a native function[32]. In the case of mutations that compensate for deletion mutagenesis, a debilitated variant should need to pass through a deterministic bottleneck to initiate a new quasispecies distribution. Therefore, compensation should be governed by selection for optimal viral fitness and not by stochastic drift [33].

Our findings indicate that reversion is not limited to a single trajectory. Compensatory mutations in both the untranslated leader and the gag-coding region emerged during long-term passage in different T-cell lines, and these mutations were required for full restoration of viral replication. Interestingly, the A423G substitution, located within the DIS, was shown to be active in restoring efficient levels of viral RNA packaging, while mutations in either the nucleocapsid, G31K, E18G, or within the p6 protein of Gag, G49K, were essential for the proper processing of Gag precursors. In each instance, the presence of three point mutations was functionally synergistic in regard to rescue of both viral RNA packaging and Gag processing. Moreover, the observed changes in regard to impaired Gag processing could be corrected by either the E18G, G31K or G49K mutations. We also showed that RNA dimerization could be partially recovered due to compensatory mutations in NC. Several studies have shown the interplay that exists between viral RNA and viral proteins that are involved in regulation of core structure, proteolytic processing, and maturation of RNA dimers [34,35]. Interestingly, SD2 mutants harboring A423G and various combinations of K197R and G49K did co-package a high molecular weight RNA species reminiscent of the "immature" dimer found in protease mutants of MLV [11].

Numerous studies on HIV-1 have shown that NC is the major protein domain within Gag that recognizes the encapsidation signals present within leader sequences [36]. The NC protein contains two zinc finger motifs that contribute to its specific interactions with viral RNA, including a well-described role in RNA dimer maturation [19,37]. Deletions within SL1 of HIV-1 were shown to impair viral replication, as well as to cause delayed processing of Gag proteins and decreased levels of packaging of viral RNA [38]. Forced evolution of SL1 deleted virus in HIV-1 showed that compensatory reversion was a result of substitutions in four disparate regions of Gag, i.e. NC (T24I), MA (V35I), CA (T24I) and p2 (T21I) [22]. These substitutions all involve hydrophobic amino acids. In contrast, we have shown that the deletions in leader sequences of SIV_mac239 _can be rescued by compensatory point mutations elsewhere within the DIS and Gag. The present work shows that restoration of SIV replication involved two distinct sets of mutations, located in both the DIS loop (A423G) and within different Gag proteins, i.e. NC (E18G and G31K) or CA (K197R) and p6 (E49K); these amino acid changes, with the exception of K197R, result in a net increase in the number of positively charged residues within Gag.

The finding that mutations within NC can rescue these deficits further confirms the role of this protein in interactions between Gag and RNA leader sequences of SIV, which have been less intensively studied than for HIV. The debilitated SD2 virus may be able to correct the deficit caused by the deletion within the DIS stem by altering both the leader sequence, as well as by reconfiguring Gag proteins, presumably to facilitate both viral RNA-RNA and RNA-protein interaction [39,40].

Our data also show that p6 plays an important role in the incorporation of viral proteins into virions and the specific encapsidation of viral RNA [41]. We have also demonstrated that a substitution within p6 resulted in comparable levels of compensation as did mutations within NC, i.e. E18G or G31K, in restoration of Gag processing. This suggests that p6 may also be important at core positioning and condensation during viral budding. The multimerization of Pr^55 ^Gag has been shown to occur on an RNA scaffold, and encapsidation of viral RNA likely requires that leader RNA sequences exist within the constraints of proper tertiary structure, which are highly conserved in both HIV-1 and SIV [40,42-44]. Deletions of leader sequences may alter critical RNA-protein interactions at early stages of viral assembly, thereby altering morphogenesis. As a result, nascent particles may not be able to undergo a "normal" intra-virion transition that condenses the RNA genome and multiple viral proteins to produce a "primed" infectious core [39].

These observations suggest the importance of functional interactions between Gag-proteins and the RNA-leader in both HIV-1 and SIV, but also imply that important differences may exist between SIV and HIV-1 in regard to such interactions. We have also demonstrated that different cell types can reproducibly select for different sets of compensatory mutations, but that both of these sets are functionally interchangeable in regard to their ability to restore viral replication, regardless of the cell type in which the virus is ultimately grown. Of course, it is conceivable that either the same mutational spectrum or even different ones may have been observed in either of the cell lines tested had additional replication studies been performed.

It is not trivial that the mechanisms of compensation for lentiviruses, grown under conditions of stress as demonstrated here, are apparently not restricted to single pathways. The mechanisms behind viral escape from antibodies, cytotoxic-T lymphocyte pressure and the generation of resistance to antiretroviral drugs are not mutually exclusive. Our results add to what is known about the plasticity and adaptability of lentivirus genomes.

## Methods

### Construction of recombinant proviral SIV clones

A PCR-based mutagenesis method was applied together with conventional cloning techniques using the full-length infectious clone of SIV, termed SIV_mac239 _wild type as a template, to generate all the mutants described [6]. All nucleotide designations are based on published sequences; the transcription initiation site corresponds to position +1 [45].

### Viral RNA packaging analysis by RT-PCR

To study packaging of viral genomic RNA we used methods previously described [6-10]. Briefly, viral RNA was isolated using the QIAamp viral RNA mini kit (QIAGEN) from equivalent amounts of 293T cell-derived viral preparations (normalized by SIV p27-CA antigen). RNA samples were treated with RNase-free DNase I at 37°C for 30 min to eliminate potential plasmid DNA contamination, followed by inactivation by incubation at 75°C for 10 min. The viral RNA samples were quantified using the Titan One Tube RT-PCR system (Boehringer Mannheim, Montreal, Quebec, Canada). The primers sg1 and sg2 were used to amplify a 114-bp fragment within the MA coding region of gag representing full-length viral RNA. The primer sg2 was radioactively labeled with δ-P^32^-ATP in order to visualize PCR products. Equivalent RNA samples, based on p27 antigen levels, were used as templates in an 18-cycle RT-PCR. The products were fractionated on 5% polyacrylamide gels and exposed to X-ray film. Relative amounts of products were quantified by molecular imaging (BIO-RAD Imaging). RNA encapsidation was determined on the basis of four different reactions, and calculated with wild type virus levels arbitrarily set at 1.0.

### Non-denaturing Northern analysis

Culture fluids from transfected 293T cells were collected and clarified using a Beckman GS-6R bench centrifuge at 3,000 rpm for 30 min at 4°C. Viral particles were further purified through a 20% sucrose cushion at 40,000 rpm for 1 hour at 4°C using a SW41 rotor in a Beckman L8-M ultracentrifuge. Viral pellets were first dissolved in Tris-EDTA (TE) buffer, then in lysis buffer containing proteinase K (100 μg/ml) and yeast tRNA (100 μg/ml). Samples were incubated for 20 min at 37°C, in the presence of 50U of DNAse I, followed by two extractions, first in phenol: chloroform: isoamyl alcohol, then chloroform. Viral RNA was then precipitated, washed in 70% ethanol and stored at -80°C until required, at which time samples were resuspended in TE buffer at 4°C. RNA was then analysed by non-denaturing electrophoresis on 0.9% agarose gels in 1× Tris-Borate-EDTA (TBE) running buffer for 4 hrs at 4°C. Products were subsequently denatured in 50 mM NaOH and equilibrated in 200 mM Na-acetate. Following electrophoresis, RNA was transferred to Hybond-N nylon membranes by capillary blotting using a 20× concentration of SSPE buffer. Membranes were baked for 2 hrs at 80°C. Probes were prepared by digestion and purification of the NdeI-BstE III fragment excised from the SIV_mac239 _plasmid. These were recovered by gel purification and labelled with δ-P^32^-ATP by nick-translation following standard protocols (Roche, Indianapolis, IN, USA). The denaturing Northern analysis of cellular RNA was also conducted in parallel. RNA extraction was carried out in similar fashion to that described for slot blotting above. Cellular RNA from lysates was normalized on the basis of p27-CA antigen present in cellular lysates. Total cellular RNA preparations, i.e. equivalent volumes of RNA, were also run on 1% ethidium bromide (EtBr) stained gels as internal controls for total RNA and 28S and 18S ribosomal RNAs. Probes were prepared as described above. Probes were labelled by nick-translation following standard manufacturer's protocols (Roche, Indianapolis, IN, USA) and used in standard hybridization reactions.

### Western analysis of viral protein

At 48 hrs post-transfection, virus-containing supernatants recovered from transfected 293T cells were collected and clarified at 3000 rpm for 30 min, at 4°C in a GS-6R Beckman centrifuge. Virus was further purified by pelleting through a 20% sucrose cushion by ultracentrifugation at 35000 rpm in a Beckman ultracentrifuge for 1 hr at 4°C. Cells were washed 2× in cold PBS and lysed by the addition of buffer containing 1% Nonidet P-40, 50 mM Tris-CL (pH 7.4), 150 mM NaCl, 0.02% sodium azide, and a cocktail of protease inhibitors (Roche, Laval, Quebec, Canada). Virus was normalized on the basis of p27-CA protein present in supernatants or cell lysates. Both pelleted virus and cellular lysates were subject to Western blotting with monoclonal antibodies directed at SIV p27-CA antigens (Fitzgerald industries, MA, USA) following standard protocols [46].

#### Cell culture and preparation of virus stocks

293T cells were maintained in DMEM medium supplemented with 10% heat-inactivated fetal bovine serum, penicillin, streptomycin and glutamine. CEMx174 or C8166 cells were maintained in RPMI-1640 medium supplemented with 10% heat-inactivated fetal bovine serum and antibiotics. All media and sera were purchased from Gibco inc. (Burlington, Ontario, Canada).

Monkey peripheral blood mononuclear cells (PBMCs) were isolated from the blood of healthy rhesus macaques (*Macaca mulatta*) housed at L.A.B. Pre-Clinical Research International Inc., (Montreal, Quebec). All primates were housed in accordance with accredited laboratory care standards. All donor macaques were tested serologically and were negative for simian type-D retrovirus-1 (SRV-1), simian T-cell lymphotrophic virus type 1(STLV-1), and simian foamy virus (SFV-1) at the initiation of the study.

PBMCs were purified on Ficoll cushions, washed in supplemented RPMI-1640 media, and purified lymphocytes were then phytohemagglutinin (PHA)-stimulated for 3 days, then maintained in supplemented RPMI-1640 medium containing 10% heat-inactivated fetal bovine serum and 20 u/ml IL-2 at 37°C with 5% CO_2 _overlay. All recombinant viral constructs were purified using a maxi-plasmid purification kit (Qiagen inc. Mississauga, Ontario, Canada). For the production of infectious viral stock, 293T cells were transfected using the above constructs together with Lipofectamine-Plus reagent (Gibco, Burlington, Ontario, Canada). Virus-containing culture supernatants were harvested at 48 hr post-transfection and clarified by centrifugation for 30 min at 4°C at 3,000 rpm in a Beckman GS-6R centrifuge. Viral stocks were passed through a 0.2 μm filter and stored in 1 ml aliquots at -80°C. All wild type and mutant stocks were titered on the basis of p27-CA antigen in culture supernatants using a Coulter SIV core antigen ELISA assay (Immunotech inc., Westbrook, ME, U.S.A.).

### Virus replication in macaque donor PBMCs

To initiate infection, viral stocks were thawed at room temperature. Then, 100 U of Dnase I in the presence of 10 mm MgCl_2 _were added at 37°C for 0.5 h to eliminate any potential plasmid DNA contamination, prior to inoculation of cells. Infection of rhesus PBMCs was performed by incubating 4 × 10^6 ^PHA-activated cells with wild type or mutant viral stocks containing 10 ng of p27-CA viral equivalent at 37°C for 2 hours. Infected cells were then washed three times with PBS to remove any remaining virus. Finally, cells were resuspended in fresh supplemented RPMI-1640 medium. Cells were maintained in 3 ml of culture medium as described above, and fresh stimulated PBMCs were added to the cultures at weekly intervals. Virus production in culture fluids was monitored by both RT assay and SIV p27 antigen capture assay.

Virus infectivity (TCID_50_) was determined by infection of CEMx174 cells as described previously. TCID_50 _results were calculated by the method of Reed and Muench [47].

### Electron microscopic analysis of virion morphology

Viral ultra-structure for the described mutant viruses was examined by transmission electron microscopy. Briefly, COS-7 cells transfected with wild type or mutant SIV constructs were fixed at 48 hours post-tranfection in 2.5% glutaraldehyde/phosphate buffered saline followed by a secondary fixation of lipids in 4% osmium tetroxide. Samples were routinely processed and serially dehydrated. Samples were embedded in Epon under vacuum followed by heat-induced polymerization. Thin-sectioned samples were stained with lead citrate and uranyl acetate and visualized at 80 Kev using a JEOL JEM-2000 FX transmission electron microscope equipped with a Gatan 792 Bioscan wide-angle 1024 × 1024 byte multi-scan CCD camera. At least 100 viral particles were scored for each variant to determine the relative percentage of particles with structural anomalies.
